# Patients’ Perceptions of Safety in Primary Healthcare Settings: A Cross-Sectional Study in the Qassim Region of Saudi Arabia

**DOI:** 10.3390/healthcare11152141

**Published:** 2023-07-27

**Authors:** Ibrahim Alasqah

**Affiliations:** Department of Public Health, College of Public Health and Health Informatics, Qassim University, Albukairiyah 52741, Saudi Arabia; i.alasqah@qu.edu.sa; Tel.: +966-1630-20518

**Keywords:** patient safety, primary care, safety experiences, harms, Saudi Arabia

## Abstract

This study assessed patients’ perceptions of safety and experiences in primary healthcare in the Qassim region of Saudi Arabia. Between July and September 2022, 730 patients from primary healthcare centers were surveyed using a multi-staged cluster random sampling approach. The Patient-Reported Experiences and Outcomes of Safety in Primary Care (PREOS-PC) questionnaire was used to measure patients’ perceived safety and experience in primary healthcare settings within the past year. Descriptive analyses were performed to report patients’ perceived safety experiences. The statistical analysis examined individual items and scales. A considerable proportion of patients reported encountering safety problems, ranging from 11% (vaccine-related) to 27% (diagnosis-related). Diagnostic errors were the most common perceived safety problem (26.7%), followed by communication issues (24.1%) and medication errors (16.3%). Between 26% and 40% experienced harm, including financial problems (40%), increased care needs (32.4%), physical health issues (32%), limitations in activities (30.6%), increased healthcare needs (30.2%), and mental health concerns (26.8%). Patient-reported safety experiences reported in our study offer valuable insights into primary care safety in Saudi Arabia. Collecting routine patient feedback is crucial for addressing identified safety problems and implementing standardized procedures.

## 1. Introduction

Globally, unsafe medical practices cause injury, disability, and death to millions of patients annually. Patient safety has gained wider recognition over the past decade, and the involvement of patients in the prevention or reduction of adverse events and harm has shown potential benefits [1,2,3,4]. The World Health Organization (WHO) defines patient safety as ‘the prevention of errors and adverse effects on patients associated with healthcare’ and ‘to do no harm to patients’ [5]. Patients can provide diverse perspectives on adverse events, and their observations play a crucial role in the planning and delivery of safe and effective healthcare [6,7,8,9,10]. Based on patient-reported information, effective strategies can be implemented to make healthcare systems safer for patients. However, most studies on patient-reported safety incidents have been conducted in hospital settings, with little attention paid to primary healthcare (PHC).

Given the heterogeneity of PHC services, the WHO (2016) suggests that service users, as the only constant factor along the healthcare pathway, are well placed to provide information that can significantly contribute to improving safety [4]. Few studies have tried to describe the nature and frequency of patient-reported safety incidents or harm in PHC in developed countries. The frequency of safety incidents or harm reported in these studies ranged from 7% to 45% [11,12,13,14,15]. The variations in reporting in these studies could be due to differences in the methods, definitions, and measurements of safety incidents in primary care. Studies examining the level of harm during the treatment process showed that harm occurred in between 7% and 29% of the patients [12,13,14,15]. One study reported severe harm to patients from diagnostic procedures (41.7%) and treatment errors (45.7%) [16]. Few studies have examined the various contributory factors to safety incidents reported by patients in primary care [14,15,17,18,19,20,21,22]. The major contributory factors that appeared in these studies included patient-healthcare provider relationships, communication with patients, accessibility of care, teamwork, organization of care, continuity of care, dignity and respect, patient characteristics, safety culture, the flow of information, and non-adherence to medication by patients. These factors play a significant role in determining patient priorities and perspectives on safety in primary care. Other factors contributing to poor patient safety in primary care settings include errors in diagnosis, unsafe medication practices, fragmentation of care, poor access to patient histories, inadequate clinical experience, age-related issues, and increased staff workload.

In developing countries, the growing importance of patient safety in PHC is epitomized by the Safer Primary Care Initiative set up by the WHO in 2012 to improve knowledge and awareness of many risks to patients, the nature and magnitude of preventable harm caused by unsafe practices, and to implement safer initiatives to protect patients [23]. However, there is little evidence of patient-reported safety problems in PHC in developing countries. Although some literature exists, it focuses on patients’ concerns about quality of care rather than safety perspectives. A review of studies conducted in 21 countries at different points in time showed that 2–3 patient safety incidents occurred every 100 primary care visits [24]. Existing evidence also suggests that 45% to 76% of unsafe incidents are preventable [25]. Despite improvements in awareness of unsafe primary care for population health, there is limited evidence of various measures that can be adopted to improve patient safety in primary care in developing countries. Most studies in this field have considered patient safety from the healthcare provider’s perspective, with too little attention given to patient perspectives [15,16].

In the Kingdom of Saudi Arabia (KSA), patient safety has been considered one of the critical elements for delivering quality healthcare, and under Saudi Vision 2030, several initiatives have been undertaken to address patient safety issues. The health sector is undergoing swift reforms under the National Transformation Program (NTP) as part of its vision, which emphasizes improving access, quality, and efficiency of healthcare services and promoting health risk protection. To achieve these goals, the Kingdom has shifted its focus from investing in tertiary and secondary healthcare facilities to restructuring and strengthening PHC facilities. Health reforms primarily focus on tackling the increasing burden of non-communicable diseases by prioritizing PHC centers as the core of the new model of healthcare delivery. A national project on hospital surveys of patient safety culture was launched in July 2017 to assess and improve the patient safety culture of healthcare organizations. The Saudi Patient Safety Center was set up to fulfill one of the initiatives of the vision: to galvanize all stakeholders, such as health policymakers, regulators, payers, healthcare providers, patients, families, and communities, about patient safety to provide health services free from harmful events.

Despite the policy focus on patient safety in the KSA, there have been an increasing number of medical complaints and claims against healthcare providers due to mortality and morbidities related to adverse events [26,27,28,29,30]. However, approximately 91% of the events reported between 2012 and 2015 were categorized as preventable errors [29]. Some hospital-based studies on patient safety culture in the Kingdom have found many contributing factors for poor patient safety, such as blame culture and poor communication due to language barriers, diversity, and cultural differences in the health workforce [31,32], barriers to reporting medication errors, poor leadership, poor communication, and an inadequate health workforce [28]. Studies have also identified factors that positively contribute to patient safety cultures, such as supportive organizational attitudes, effective teamwork, management support, effective leadership and supervision, support from other departments, organizational learning, and continuous improvement of health staff [28,33,34]. A few studies on safety culture in primary care have highlighted the importance of stress recognition and openness in communication as steps toward preventing adverse events [35,36].

Most studies conducted in KSA have explored the impact of safety culture and health service quality from the perspective of healthcare providers. However, studies examining patient safety issues from the patient’s perspective are scarce [28]. Moreover, there is a significant gap in knowledge about patient-reported safety incidents in PHC in the KSA. This study examined patient-reported experiences and outcomes of patient safety in primary care in the Qassim region of the KSA.

## 2. Materials and Methods

### 2.1. Measurement of Patient-Reported Experiences

Patient experiences and satisfaction with the use of primary care services are fundamentally distinct from their perceptions of safety. In earlier studies, patient feedback on safety in PHC was collected as part of a patient satisfaction survey. Recently, few instruments have been available to measure patient safety in primary care, and they have mostly focused on the outcomes of safety events in primary care settings. This study used patient-reported experiences and outcomes of safety in primary care (PREOS-PC), developed by Cabello et al. [15]. This validated instrument provides a comprehensive measure of the opinions, experiences, and safety outcomes of patients in PHC. Moreover, it is a multidimensional instrument for measuring patient-reported experiences and safety outcomes in primary care settings.

### 2.2. Study Design

This was a cross-sectional study of patients who attended PHC centers of the Ministry of Health (MOH) in the Qassim region, KSA, between July and September 2022. All patients aged >18 and <65 years, speaking Arabic (including parents in the case of children), who visited PHC centers in the Qassim region were included in the study. Non-Arabic speakers and patients with severe illnesses, including mental health issues, were excluded from the study.

### 2.3. Sample Size

According to a 2021 Ministry of Health (MOH) report [37], 155 PHC centers are found across four geographical zones of the Qassim region: North, South, East, and West. Data were collected from a total of 800 patients (200 from each region) from all PHC centers in the region on a random basis. The required sample size was calculated using the EpiInfo^TM^ application. Cluster random sampling (disproportionate) was deemed appropriate for this study. The minimum required sample size was 384 (96 from each cluster) for a 95% confidence interval, 50% expected frequency, 5% margin of error, and design effect 1. However, as a safeguard against a low response rate, it was decided to recruit 200 cases in each cluster.

### 2.4. Sample Selection

A multistage random sampling method was used to select the required number of patients attending services in PHC centers. In the first stage, PHC centers in the Qassim region were divided into four groups according to their geographic locations: east, west, north, and south. Ten PHC centers were selected from each geographic location using a simple random technique, resulting in a total of 40 PHC centers from all locations. The second stage involved recruiting equal numbers of patient participants from the selected PHC centers, and 20 patients from each center were invited to take part in the study by filling out the study questionnaire.

These patients were selected by consecutive sampling until the required number of samples were obtained from each PHC center. Data collection from all selected PHC centers was completed on 28 September 2022. After scrutinizing the completed questionnaires and removing those with incomplete responses, 730 questionnaires were considered for the analysis. 

### 2.5. Data Collection Instrument

In this study, a simplified version of the validated PREOS-PC questionnaire translated into Arabic was used. The version used in this study had several items distributed across four main domains: practice activation (what a practice does to create a safe environment and ensure safety), patient activation (how proactive patients are in ensuring safe healthcare delivery), experiences of safety problems (safety errors), and outcomes of patient safety (harm). The questionnaire had 27 questions, including practice evaluation (ten questions), safety perceptions (two questions), safety problems (nine questions), and harm related to safety incidents (six questions). 

The practice evaluation questions were related to the availability of doctors, consultation time, communication, and access to information. Safety perception questions are related to the safety priorities and trustworthiness of providers. Experiences of safety problems included questions related to prescription, diagnosis, communication between staff, and health information. Experiences of harm included questions on mental health, restriction of activities, physical health, other treatment, financial needs, and the need for personal care. The survey questionnaire was designed to measure patient-reported safety incidents. Respondents reported their perceptions, experiences, and outcomes about the safety of healthcare received from PHC centers over the previous 12 months. 

### 2.6. Data Collection

Data collection was conducted between 4 July and 28 September 2022, by the research team. Adequate care was taken to ensure the privacy and confidentiality of participants and family members while providing information. Data were collected only after obtaining written, informed consent from respondents. The survey questionnaire was distributed to the patients after explaining the aim of the study. All completed questionnaires were verified for completeness and confirmed by research investigators who were available at the time of data collection to clarify any inquiries.

### 2.7. Statistical Analysis

The collected data were verified, coded, and entered into a personal computer. SPSS (Statistical Package for the Social Sciences) software version 26 was used for the data analysis. Statistical analysis was conducted at the patient level and was based on individual items and scales, which consisted of the calculation of the number and percentage of patients answering each response category for each item. The scale scores were calculated as a percentage of the maximum achievable score for all items, with scores ranging from 0 to 100. For all scales, higher scores indicate higher levels of patient safety. 

### 2.8. Ethical Approval 

This study was approved by the Regional Research Ethics Committee, Qassim Province, KSA. Permission was also obtained from the directors of the selected PHC centers. Informed consent was obtained from all the participants.

## 3. Results

Table 1 provides information on the demographic characteristics of the study participants, including sex, age, marital status, and education level. Most participants were male (57.3%), while 42.7% were female. In terms of age, the largest group was in the 20–29-year range (60.7%). Most participants were unmarried (73.0%). Regarding education, most participants had a bachelor’s degree or higher (58.9%).

Table 2 sheds light on patients’ evaluations of practice at PHC centers based on various criteria. The columns in the table show the percentage of responses for “Never”, “Rarely”, “Sometimes”, “Often”, and “Always” about different aspects of the doctor’s performance, such as availability, communication, addressing concerns, explaining tests/treatments, informing concerning side effects, taking patients’ concerns seriously, helping with care arrangements, access to information, awareness of recommendations from other professionals, and working well with other staff in the PHC centers. The highest response (percentage) for each criterion indicates the level of evaluation that patients rated the highest, ranging from “Always” to “Never”. For example, the highest response for “Doctor available when needed” was “Always”, with 40.4% of patients providing this rating. Similarly, the highest response for “Doctor gave the patient enough time to say and ask questions” was “Always”, with 54.5% of patients giving this rating. This information provides useful insights into patients’ perceptions of the performance of primary care doctors against different criteria.

Table 2 further stands for patients’ evaluation of their perception of practice at PHC centers based on two criteria: “Delivering safe care was a top priority for the practice in the PHC centers” and “Healthcare workers in the PHC centers were trustworthy”. For the criterion “Delivering safe care was a top priority for the practice in the PHC centers,” the highest response was “Agree”, with 35.3% of patients giving this rating, followed by “Strongly agree”, with 39.0% of patients giving this rating. For the criterion “Healthcare workers in the PHC centers were trustworthy”, the highest response was “Agree”, with 35.6% of patients giving this rating, followed by “Neither agree nor disagree”, with 23.2% of patients giving this rating.

Table 3 indicates which aspects of primary care practice patients perceive to be associated with safety problems. The combined responses of “more than once” and “only once” highlight areas where patients reported experiencing safety problems in PHC, while the “never” responses stand for areas where patients reported not experiencing safety problems. The findings showed that patients reported experiencing safety problems in different areas of PHC, such as diagnosis (26.7%), medication (16.3%), other non-pharmacological treatments (11.3%), vaccines (10.9%), blood tests and other laboratory tests (11.7%), diagnosis and monitoring procedures (12.3%), patient-provider communication (24.1%), and health records (13.6%). Most patients reported never experiencing safety problems in these areas, ranging from 53.6% (diagnosis) to 76.0% (blood tests and other laboratory tests). However, a notable percentage of patients responded with “do not know,” indicating uncertainty about their perception of safety problems.

These findings suggest that efforts should be made to address patient safety concerns, particularly in areas in which patients report experiencing safety problems. Patient-provider communication had the highest percentage (24.1%) of patients reporting safety problems, indicating a potential area for improvement in PHC. Health records had the lowest percentage (13.6%) of patients reporting safety problems. However, it is important to note that a significant percentage of patients responded “do not know,” indicating the need for improved patient education and communication about patient safety in PHC settings.

Table 4 presents patients’ responses concerning their experiences of harm in PHC settings over the past 12 months. The most common types of harm were financial issues (40.7%), more personal care needs (32.4%), physical health problems (32.1%), limitations in performing usual activities (30.7%), and increased healthcare needs (30.1%). Regarding mental health, anxiety, or stress, 26.8% of the patients reported experiencing harm “a lot” or “somewhat”, while 73.2% reported experiencing harm “not at all” in terms of mental health, anxiety, or stress in PHC settings in the past 12 months. In terms of limitations in performing usual activities in PHC settings in the past 12 months, 30.7% of patients reported experiencing harm “a lot” or “somewhat”, while 69.3% reported experiencing harm “not at all”. In the physical health domain, 32.1% of patients reported experiencing harm “a lot” or “somewhat”, while 67.9% reported experiencing “not at all” harm in terms of physical health in PHC settings in the past 12 months. In terms of increased healthcare needs, 30.2% of patients reported experiencing harm “a lot” or “somewhat”, while 69.9% reported experiencing harm “not at all” in PHC settings in the past 12 months. Financial: 40.7% of patients reported experiencing harm “a lot” or “somewhat”, while 59.3% reported experiencing harm “not at all” in terms of financial harm in PHC settings in the past 12 months. Increased personal care needs: 32.4% of patients reported experiencing harm “a lot” or “somewhat”, while 67.7% reported experiencing harm “not at all” in terms of increased personal care needs in PHC settings in the past 12 months.

The table shows the proportion of patients who reported experiencing “a lot” or “somewhat” of harm in various aspects of PHC, as well as the proportion of patients who reported experiencing “not at all” of harm. This highlights that a significant percentage of patients reported experiencing harm in certain areas, such as financial harm, while others reported little to no harm in terms of mental health, physical health, and personal care needs. These findings underscore the need to address patient safety and well-being in PHC settings, particularly in areas where patients experience significant harm, and continuously strive to improve their provision of safe and high-quality care.

## 4. Discussion

The study revealed that most patients had a positive experience with safety practices at PHC centers. However, a considerable proportion of patients in the study reported safety problems once or more than once, ranging from 11% for vaccine-related safety problems to 27% for diagnosis-related problems. The study also showed that 26% (mental health-related) to 40% (financial issues) of patients reported experiencing a certain degree of harm because of the services received from the PHC center in the previous 12 months. One of the major findings of this study is that the proportion of patients who reported experiences of safety problems was much higher than that in earlier studies in other countries by Solberg et al. (5.5%) [12] and Kistler et al. (15.6%) [16]. However, this proportion is lower than the findings of an English study by Ricci-Cabello et al., who reported that at least 45% of patients experienced at least one safety problem with PHC received in the previous 12 months [15]. 

The high rate of safety problems seen in this study was related to ‘diagnostic errors,’ as reported by 26.7% of the patients. This percentage is much higher than the 17% rate reported by Ricci-Cabello et al. in England [15] and the 13% rate reported by Kistler et al. in the United States [16]. Diagnosis is an important task performed in PHC centers, but diagnosis is often missed, delayed, or wrongly reported, which often leads to an aggravation of patients’ health problems or results in a different diagnosis after seeking further opinion. As the first point of contact and the most accessible place for treatment, PHC centers often face large patient volumes, and diagnosis becomes a high-risk area for errors [38]. Diagnostic errors in PHC are more common than other types of errors, but their incidence is unknown. Numerous studies have estimated that between 5% and 15% of preventable harms are due to diagnostic errors, and these percentages vary depending on the type of PHC setting and methods used in earlier studies [39]. A study in Sweden of reported cases of preventable harm in primary care found that 46% were due to diagnostic errors [40].

The second most frequent safety problem was related to patient-care provider communication, reported by 24.1% of the respondents. Several studies in the KSA have highlighted the issue of communication barriers between patients and health service providers [41], particularly with expatriate nursing staff, who constitute approximately 61% of all nursing staff in the Kingdom [42]. Patient-centered interactions with health service providers are considered a prerequisite for the delivery of safe health services. Owing to a lack of knowledge of the Arabic language, they rely on non-verbal cues, which often result in misinterpretations of the meaning of communication. Many expatriate nurses who have little knowledge about the culture and religious practices of patients may experience problems understanding health problems, leading to adverse effects on the nurse-patient relationship and patient safety [33,34,43]. Language diversity among healthcare service providers causes several challenges in patient care, including life-threatening conditions, particularly when taking the history of the patient during the delivery of healthcare services. Studies have shown that barriers in communication between patients and health service providers due to differences in cultural and religious practices have adverse consequences for patient safety in the domains of medication management and the psychological, emotional, and physical wellbeing of patients and their family members [6,31]. In some cases, medication errors are underreported by expatriate nurses because of fear of disciplinary action [44]. To improve effective communication between patients and health service providers, existing in-service training programs must incorporate components that cover language, cultural, and religious practices. Other measures, such as mentorship programs for expatriate nurses and facilities for online translation tools for health service providers, may also be introduced. Studies on patient-provider communication in the KSA have used the perspectives of healthcare providers, with a limited focus on patients. Therefore, future research should explore the perspectives of patients and family members concerning barriers to patient-provider communication.

The third most frequent safety problem was related to medication errors (16.3%), a failure during the treatment process that often led to potential harm to the patient. Several studies on patient safety in PHC have focused on medication errors that occur during the medication use process. Medication errors may occur at every stage of patient care, such as prescribing, dispensing, administering, and monitoring; however, the percentage of errors occurring at each stage may differ. Despite all efforts made by the government, prescription errors are still a huge concern as a cause of harm to patients. A study on medication errors in primary care in the KSA found that 18.7% of all medication errors were related to prescription errors [45]. However, another study found that 84.8% of errors were detected at the time of prescription, 5.8% at the time of transcription, and 5.7% at the dispensing stage [46]. Thus, there is an urgent need to create knowledge and address training about medication errors. In the KSA, underreporting of medication errors is a widespread problem, as revealed by a study in which 58.8% of health facilities did not report medication errors [47]. In addition to improving knowledge and awareness through CPD training on prescription errors, a collaborative approach should be facilitated between professionals such as physicians, nurses, and pharmacists. Pharmacists could perform medication reconciliation and review, thus working collaboratively within multidisciplinary teams in order to achieve the responsible use of medicines across primary care settings.

A considerable proportion of the patients in this study reported some degree of harm after the use of primary care services in the previous 12 months. Patients who attended PHC centers were more likely to report being harmed financially (40%); increased personal care needs (32.4%); physical health issues (32%); limitations in performing usual activities (30.6%); increased healthcare needs (30.2%); and mental health, anxiety, or stress (26.8%) because of using primary care in the previous 12 months. Notably, these harms were significantly higher than those reported in earlier studies [15,48]. This higher prevalence of harm might be an underestimation attributable to how patients conceptualize safety and harm in the context of the KSA. The findings might have been affected by respondents’ recall bias because the data were collected for the previous 12 months. Nevertheless, this is the first quantitative study to measure patient-reported safety experiences in primary care in KSA. The study revealed that there is a substantial burden of avoidable harm in primary care, which is mostly attributable to diagnostic errors, patient-provider interactions, and medication-related errors.

## 5. Conclusions

This study is the first examination of patient-reported experiences and outcomes regarding the safety of PHC in the KSA. The findings highlight priority areas for improving safety practices in PHC settings, specifically physician appointments, health provider-patient interactions, and safe delivery practices. The study reveals that common patient safety issues related to diagnosis, patient-provider interactions, and medication practices are prevalent and preventable across various healthcare systems. However, the existing safety practices in PHC settings are still unclear, potentially due to a discrepancy between healthcare providers’ practices and patients’ feelings regarding safety concerns. To enhance patient safety in primary care, the routine collection of patient-reported safety data using standardized and validated instruments is crucial. By using patient feedback, standardized procedures can be developed and implemented to promptly address safety events and concerns, fostering a patient-centered approach to improving safety practices in PHC settings in the KSA. 

## Figures and Tables

**Table 1:** Socio-demographic information of the participants.

Variables	Participants	
	Frequency	Percent
**Sex**		
Male	418	57.3
Female	312	42.7
**Age**		
<20 years	128	17.5
20–29 years	443	60.7
30–39 years	77	10.5
40–49 years	48	6.6
50–59 years	26	3.6
>59 years	8	1.1
**Marital status**		
Married	182	24.9
Unmarried	533	73.0
Widow	5	0.7
Divorced	10	1.4
**Education**		
No formal education	2	0.3
Primary or Intermediate	26	3.6
Secondary	174	23.8
Diploma	98	13.4
Bachelor or higher	430	58.9

**Table 2:** Patient’s Evaluation of Practice at PHC Centers.

Patient’s Evaluation of Practice at PHC Centers	Percent				
	Never	Rarely	Sometimes	Often	Always
Doctors are available when needed	1.0	3.8	21.6	33.2	40.4
The doctor gave the patient enough time to say something and ask questions	0.7	3.4	14.5	26.8	54.5
The doctor encouraged the patient to talk about healthcare concerns	4.2	8.9	16.6	24.4	45.9
The doctor explained tests/treatments to the patient	2.1	7.0	14.5	25.5	51.0
The doctor told the patient about the side effects	15.6	19.5	20.1	13.7	31.1
The doctor took the patient’s concerns seriously	5.5	9.5	24.0	23.7	37.4
The doctor helped arrange/organize the right type of care	4.4	6.6	19.3	25.8	44.0
The doctor had access to information when needed	3.4	5.6	16.8	22.7	51.4
The doctor was aware of the recommendations from other professionals	8.5	8.5	23.0	25.9	34.1
The doctor worked well with other staff in the PHC	1.2	3.3	14.0	31.2	50.3
	Strongly disagree	Disagree	Neutral	Agree	Strongly agree
Delivering safe care was a top priority for the practice in the PHC	1.5	3.6	20.5	35.3	39.0
Healthcare workers in the PHC were trustworthy	1.1	5.8	23.2	35.6	34.4

**Table 3:** Patients’ perceived safety problems in the previous 12 months.

Safety Problems	Responses in Percentage			
	More than Once	Only Once	Never	Do Not Know
Diagnosis	9.9	16.8	53.6	19.7
Medication	6.6	9.7	68.8	14.9
Other (non-pharmacological) treatments	4.2	7.1	71.0	17.7
Vaccines	4.7	6.3	73.4	15.6
Blood tests and other laboratory tests	4.4	7.3	76.0	12.3
Diagnosis and monitoring procedures	5.3	7.0	72.2	15.5
Patient-provider communication	14.1	10.0	68.1	7.8
Health record	7.0	6.6	75.9	10.5

**Table 4:** Patients’ experience of harm in the previous 12 months.

Type of Harm	Response in Percentage		
	A Lot	Somewhat	Not at All
Mental health, anxiety, or stress	7.5	19.3	73.2
Limitations to doing usual activities	5.6	25.1	69.3
Physical health	8.1	24.0	67.9
Increased healthcare needs	7.3	22.9	69.9
Financial	11.5	29.2	59.3
Increased personal care needs	7.3	25.1	67.7

## Data Availability

The data that supports the findings of this study are not publicly available because I did not ask participants to consent to raw data sharing outside of the research team. Public sharing of the data could compromise anonymity and research participant consent.
